# Relative effectiveness of bivalent Original/Omicron BA.4-5 mRNA vaccine in preventing severe COVID-19 in persons 60 years and above during SARS-CoV-2 Omicron XBB.1.5 and other XBB sublineages circulation, Italy, April to June 2023

**DOI:** 10.2807/1560-7917.ES.2023.28.32.2300397

**Published:** 2023-08-10

**Authors:** Massimo Fabiani, Alberto Mateo-Urdiales, Chiara Sacco, Maria Cristina Rota, Emmanouil Alexandros Fotakis, Daniele Petrone, Martina Del Manso, Andrea Siddu, Paola Stefanelli, Antonino Bella, Flavia Riccardo, Giovanni Rezza, Anna Teresa Palamara, Silvio Brusaferro, Patrizio Pezzotti, on behalf of the Italian Integrated Surveillance of COVID-19 study group and of the Italian COVID-19 Vaccines Registry group

**Affiliations:** 1 Department of Infectious Diseases, Istituto Superiore di Sanità, Rome, Italy; 2 European Programme on Intervention Epidemiology Training (EPIET), European Centre for Disease Prevention and Control, Stockholm, Sweden; 3 General Directorate of Prevention, Italian Ministry of Health, Rome, Italy; 4 Office of the President, Istituto Superiore di Sanità, Rome, Italy; 5 Members of the author groups are listed under Acknowledgements

**Keywords:** COVID-19, bivalent mRNA vaccines, effectiveness, Omicron XBB sublineages, Italy

## Abstract

During predominant circulation of SARS-CoV-2 Omicron XBB.1.5 and other XBB sublineages (April–June 2023), we found that a second or third booster of Comirnaty bivalent Original/Omicron BA.4-5 mRNA vaccine, versus a first booster received at least 120 days earlier, was effective in preventing severe COVID-19 for more than 6 months post-administration in persons 60 years and above. In view of autumn 2023 vaccination campaigns, use of bivalent Original/Omicron BA.4-5 mRNA vaccines might be warranted until monovalent COVID-19 vaccines targeting Omicron XBB.1 sublineages become available.

The World Health Organization (WHO) and other international health agencies, including European Centre for Disease Prevention and Control (ECDC) and European Medicines Agency (EMA), have recently published statements [1,2] on a new formulation of monovalent COVID-19 vaccines targeting the severe acute respiratory syndrome coronavirus 2 (SARS-CoV-2) Omicron (Phylogenetic Assignment of Named Global Outbreak (Pango) lineage designation: B.1.1.529) XBB.1 descendent lineages, which have been predominant globally since April 2023 [3]. However, few peer-reviewed studies have evaluated the relative vaccine effectiveness (rVE) of currently approved vaccines during XBB descendant lineage circulation [4-7]. Given the limited evidence, we conducted a study to evaluate the rVE of the Comirnaty bivalent Original/Omicron BA.4-5 mRNA vaccine (BNT162b2 mRNA, BioNTech-Pfizer) in preventing severe disease because of infections with SARS-CoV-2 Omicron XBB.1.5 or other XBB sublineages in individuals 60 years and above, who comprise the main target population for the upcoming autumn 2023 COVID-19 vaccination campaigns [8].

## Study setting and representativeness of study participants

This nationwide study is representative of all individuals 60 years and above living in Italy (n = 18,326,359), where, by 4 May 2023, 16,085,062 (88%) of this age group had received at least a first booster of an mRNA vaccine [9]. Of these, 5,602,863 (35%) had received a second booster and 505,947 (3.1%) had received a third booster. On 12 September 2022, the Comirnaty bivalent Original/Omicron BA.4-5 mRNA vaccine was approved by the European Medicines Agency (EMA) [10], thereafter becoming the most used booster vaccine in Italy, where it was primarily recommended as a booster dose for all people 60 years and above and those with risk factors (this vaccine accounts for 65% of all booster doses administered between 12 September 2022 and 4 May 2023 in persons 60 years and above) [11].

**Figure 1:**
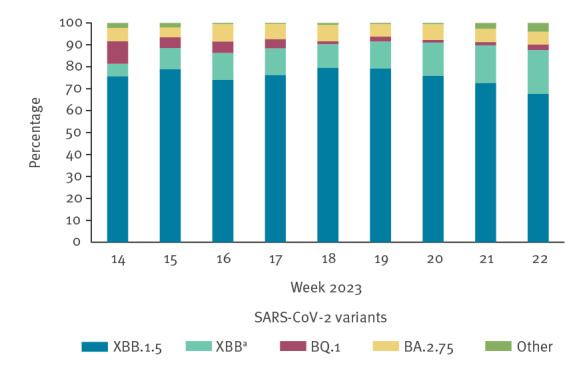
Percentage distribution of the circulating SARS-CoV-2 variants by calendar week, Italy, 3 April–4 June 2023 (n = 4,032)

We conducted a retrospective cohort study analysis using data retrieved from the national vaccination registry and the national COVID-19 surveillance system linked through the individual tax code [12,13]. The study was conducted between 3 April and 4 June 2023, a calendar period during which the Omicron XBB.1.5 and other XBB sublineages were predominant in Italy (88% in the whole study period; > 80% in each week) (Figure 1) [14,15].

SARS-CoV-2: severe acute respiratory syndrome coronavirus 2.

^a^ XBB sublineages other than XBB.1.5.

Data were extracted on 7 July 2023 to account for a 28-day time interval to ascertain any hospitalisation or death after infection and 5 days of notification delay.

**Figure 2:**
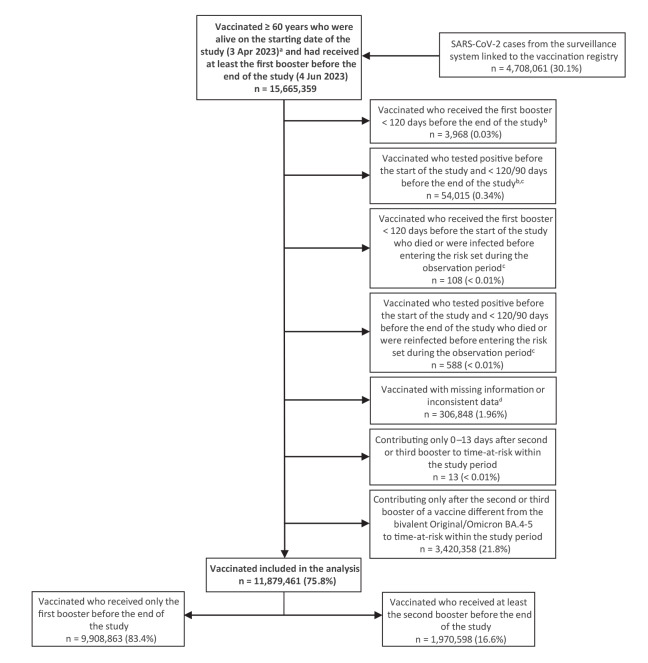
Selection of the study participants, Italy, 3 April–4 June 2023 (n = 15,665,359)

^a^ The expected date of death for causes unrelated to COVID-19 for individuals who did not receive a confirmation of SARS-CoV-2 infection during the study period was imputed according to methods described in the Supplementary Methods S1.

^b^ Not eligible for a second booster during the study period according to recommendations from the Italian Medicines Agency (booster at least 120 days since the prior vaccine administration or possible infection) [21].

^c^ Vaccinated with a second or third booster before the start of the study who were never at risk during the study period because of a prior infection less than 90 days before the end of the study (90 days was set as the minimum time interval between two consecutive infections); vaccinated with a first booster, with or without a prior infection, who died or were reinfected during the study period before becoming eligible to receive a second booster (120 days after the first booster or prior infection); or vaccinated with a second or third booster before the start of the study who had a prior infection and died before being at risk of reinfection 90 days later during the study period.

^d^ Including 33,339 (0.12%) individuals who had received a booster dose less than 120 days after a prior infection or had received two consecutive booster doses less than 120 apart (inconsistent data according to national guidelines recommending a booster dose at least 120 days since the prior vaccine administration or possible infection [21]).

We selected all 15,665,359 individuals aged 60 years and above who were alive at the start of the study (see Supplementary Methods S1 for methods used to impute the expected date of death for causes unrelated to COVID-19) and had received at least the first booster of a COVID-19 vaccine before the end of the study. We then excluded 3,785,898 (24.2%) individuals according to the criteria listed in Figure 2.

The COVID-19 vaccines used in Italy for booster vaccination were: Comirnaty monovalent Original strain mRNA vaccine (BNT162b2 mRNA, BioNTech-Pfizer); Spikevax monovalent Original strain mRNA vaccine (mRNA-1273, Moderna); Comirnaty bivalent Original/Omicron BA.1 mRNA vaccine (BNT162b2 mRNA, BioNTech-Pfizer); Spikevax bivalent Original/Omicron BA.1 mRNA vaccine (mRNA-1273.214, Moderna) and Comirnaty bivalent Original/Omicron BA.4-5 mRNA vaccine (BNT162b2 mRNA, BioNTech-Pfizer).

The baseline characteristics of the 11,879,461 individuals included in the analysis are presented in Table 1.

**Table 1:** Baseline characteristics of the individuals included in the analysis, Italy, 3 April–4 June 2023 (n = 11,879,461)

Characteristics	First booster only (≥ 120 days earlier)^a^ n = 9,908,863		Bivalent second or third booster (Original/Omicron BA.4-5)^a^ n = 1,970,598		Total n = 11,879,461	
	n	%	n	%	n	%
**Sex**						
Female	5,444,370	54.9	1,040,832	52.8	6,485,202	54.6
Male	4,464,493	45.1	929,766	47.2	5,394,259	45.4
**Age (years)**						
60–64	2,588,226	26.1	285,578	14.5	2,873,804	24.2
65–69	2,060,533	20.8	349,252	17.7	2,409,785	20.3
70–74	1,785,406	18.0	389,155	19.7	2,174,561	18.3
75–79	1,448,121	14.6	360,482	18.3	1,808,603	15.2
80–84	1,022,972	10.3	294,120	14.9	1,317,092	11.1
85–89	635,720	6.4	186,078	9.4	821,798	6.9
90–94	283,138	2.9	83,282	4.2	366,420	3.1
≥ 95	84,747	0.9	22,651	1.1	107,398	0.9
Median (IQR)	70 (64–78)		74 (68–81)		71 (65–78)	
**Country of birth**						
Italian-born	9,443,990	95.3	1,911,843	97.0	11,355,833	95.6
Foreign-born	464,873	4.7	58,755	3.0	523,628	4.4
**Geographical macroarea**						
North-west	2,345,835	23.7	693,190	35.2	3,039,025	25.6
North-east	1,786,656	18.0	384,694	19.5	2,171,350	18.3
Centre	2,042,021	20.6	496,076	25.2	2,538,097	21.4
South and islands	3,734,351	37.7	396,638	20.1	4,130,989	34.8
**High-risk group^b^**						
No	7,161,731	72.3	1,126,985	57.2	8,288,716	69.8
Yes	2,747,132	27.7	843,613	42.8	3,590,745	30.2
**Days since first booster vaccine**						
≤ 180	95,547	1.0	451	0.0	95,998	0.8
181–365	268,221	2.7	34,163	1.7	302,384	2.5
> 365	9,545,095	96.3	1,935,984	98.2	11,481,079	96.6
Median (IQR)	467 (443–485)		484 (468–503)		470 (446–488)	
**Days since prior infection**						
≤ 180	680,491	6.9	51,809	2.6	732,300	6.2
181–365	1,516,275	15.3	284,493	14.4	1,800,768	15.2
> 365	876,340	8.8	212,180	10.8	1,088,520	9.2
No prior infection	6,835,757	69.0	1,422,116	72.2	8,257,873	69.5
Median number (IQR)^c^	274 (192–378)		342 (270–415)		280 (211–382)	
**First booster vaccine^d^**						
mRNA monovalent	9,805,449	99.0	1,969,986	100.0	11,775,435	99.1
mRNA bivalent Original/Omicron BA.1	34,377	0.3	300	< 0.02	34,677	0.3
mRNA bivalent Original/Omicron BA.4-5	69,037	0.7	312	< 0.02	69,349	0.6

IQR: interquartile range; SARS-CoV-2: severe acute respiratory syndrome coronavirus 2.

^a^ Vaccinations status at the study end date.

^b^ Including residents in long-term care facilities and individuals with health risk conditions listed in Supplementary Table S1.

^c^ Among individuals who tested positive for SARS-CoV-2 infection before the start of the study.

^d^ mRNA monovalent vaccines: Comirnaty Original strain mRNA vaccine (BNT162b2 mRNA, BioNTech-Pfizer) or Spikevax Original strain mRNA vaccine (mRNA-1273, Moderna); mRNA bivalent Original/Omicron BA.1: Comirnaty bivalent Original/Omicron BA.1 mRNA vaccine (BNT162b2 mRNA, BioNTech-Pfizer) or Spikevax bivalent Original/Omicron BA.1 mRNA vaccine (mRNA-1273.214, Moderna); Comirnaty bivalent Original/Omicron BA.4-5 mRNA vaccine (BNT162b2 mRNA, BioNTech-Pfizer).

Compared with individuals who had received only a first booster at least 120 days before, those who had received at least a second booster were slightly older, more frequently living in northern-central than in southern Italy, more frequently presenting health-risk conditions, and having a longer time elapsed since a prior infection. By the end of the study period, among the 1,970,598 individuals who had received at least a second booster, 331,433 (16.8%) had also received a third booster of the bivalent Original/Omicron BA.4-5 mRNA vaccine.

## Relative vaccine effectiveness of a second or third booster 

We compared time to infection subsequently leading to severe COVID-19 (i.e. hospitalisation or death within 28 days of the swab date for a test that was SARS-CoV-2-positive) between individuals 60 years and above who had received only the first booster of an mRNA vaccine at least 120 days earlier and those who had received at least a second booster of the bivalent Original/Omicron BA.4-5 mRNA vaccine.

Cases with confirmed SARS-CoV-2 infections notified to the surveillance system include those detected through PCR or antigen test [16]. The Italian guidelines, in accordance with WHO indications [17], define a COVID-19-related death as one occurring in presence of a clinical picture suggestive of COVID-19, the absence of a clear cause of death other than COVID-19, and the absence of a complete clinical recovery from the disease. Similarly, the surveillance system is expected to record only the hospitalisations of cases presenting with clinical manifestations of the respiratory tract or other organs directly associated with SARS-CoV-2 infection.

Participants’ follow-up started either on 3 April 2023 or when they became eligible to receive a second booster or entered at risk of reinfection 90 days after a prior infection. It ended on the date of a positive test for SARS-CoV-2 infection (considered as the event date), date of death for causes unrelated to COVID-19, or 4 June 2023, whichever came first.

After splitting individual data to account for time-varying vaccination status, we used a multivariable Cox proportional hazard model to estimate the adjusted hazard ratios (HR) of severe COVID-19 according to time since administration of a second or third booster of the bivalent Original/Omicron BA.4-5 mRNA vaccine (i.e. 15–60 days, 61–120 days, 121–180 days and 181–265 days) compared with a first booster received at least 120 days earlier. Estimates were adjusted for all variables described in Table 1, further detailing the geographical area where vaccination took place (i.e. the 19 regions and two autonomous provinces of Italy). To account for differences in the calendar period of exposure between the compared groups, we used calendar time as the underlying time scale. The rVE was calculated as ((1−HR) x 100).

Compared with a first booster of an mRNA vaccine received at least 120 days earlier, the rVE against severe COVID-19 for a second or third booster of the bivalent Original/Omicron BA.4-5 mRNA vaccine in the time interval 15–60 days post-administration was 45.6% (95% confidence interval (CI): 1.6–69.9), progressively decreasing to 14.3% (95% CI: 1.6–25.3) 181–265 days post-administration (Table 2). We did not observe statistically significant differences by age group (60–79 vs ≥ 80 years; likelihood ratio test, p = 0.821).

**Table 2:** Effectiveness against severe COVID-19 of a second or third booster of the bivalent Original/Omicron BA.4-5 mRNA vaccine relative to a first booster of an mRNA vaccine received at least 120 days earlier, Italy, 3 April–4 June 2023 (n = 11,879,461)

Time since booster	Number of individuals^a^	Number of events	Rate per 100,000 PD	rVE (%)	95% CI
**Overall**					
First booster ≥ 120 days	9,912,672	3,565	0.587	Reference	
Second or third booster					
15–60 days	90,807	11	0.586	45.6	1.6 to 69.9
61–120 days	715,375	135	0.731	24.7	10.5 to 36.7
121–180 days	1,843,177	467	0.761	17.0	8.4 to 24.8
181–265 days	1,259,968	227	0.581	14.3	1.6 to 25.3
**60–79 years**					
First booster ≥ 120 days	7,884,984	1,728	0.357	Reference	
Second or third booster					
15–60 days	57,156	5	0.425	24.8	−81.1 to 68.7
61–120 days	489,537	50	0.396	28.8	5.6 to 46.4
121–180 days	1,299,074	190	0.444	14.9	1.0 to 26.9
181–265 days	901,841	96	0.337	17.9	−1.0 to 33.3
**≥ 80 years**					
First booster ≥ 120 days	2,027,688	1,837	1.488	Reference	
Second or third booster					
15–60 days	33,651	6	0.854	55.8	1.4 to 80.1
61–120 days	225,838	85	1.458	22.1	3.1 to 37.4
121–180 days	544,103	277	1.492	18.4	7.2 to 28.2
181–265 days	358,127	131	1.239	11.4	−6.1 to 25.9

CI: confidence interval; PD: person days; rVE: relative vaccine effectiveness.

^a^ The sum of the number of individuals in each time interval is greater than the total number of individuals included in the analysis because of the possible change in vaccination status during the observation period leading an individual to be counted more than once.

First booster vaccine: Comirnaty monovalent Original strain mRNA vaccine (BNT162b2 mRNA, BioNTech-Pfizer); Spikevax monovalent Original strain mRNA vaccine (mRNA-1273, Moderna); Comirnaty bivalent Original/Omicron BA.1 mRNA vaccine (BNT162b2 mRNA, BioNTech-Pfizer); Spikevax bivalent Original/Omicron BA.1 mRNA vaccine (mRNA-1273.214, Moderna) or Comirnaty bivalent Original/Omicron BA.4-5 mRNA vaccine (BNT162b2 mRNA, BioNTech-Pfizer). Second or third booster vaccine: Comirnaty bivalent Original/Omicron BA.4-5 mRNA vaccine (BNT162b2 mRNA, BioNTech-Pfizer).

## Discussion

We found that, a second or third booster of the bivalent Original/Omicron BA.4-5 mRNA vaccine, compared with a first booster of an mRNA vaccine received at least 120 days earlier, provided additional protection against severe COVID-19 during predominant circulation of the Omicron XBB.1.5 and other XBB sublineages among persons 60 years and above in Italy. Although reduced, an additional protection persisted 6 months after vaccine administration. The study was conducted during the 2023 spring season, when the number of severe cases was relatively low, compared with those generally expected during the autumn/winter season, especially in the relatively small group who had received a second or third booster recently. For this reason, the rVE estimate in the time-interval 15–60 days post-administration showed a wide 95% CI that does not allow conclusions to be made about a significant waning of protection, despite point estimates suggesting its likely occurrence. Our estimates appear slightly lower than the rVE against severe COVID-19 caused by the Omicron BA.5 subvariant previously estimated in Italy (46% vs 49–61% in the time interval 14–60 days post-administration and 25% vs 35% in the time-interval 61–120 days post-administration) [18]. However, this indirect comparison should be interpreted with caution because of the possible bias from comparing vaccine effectiveness over the same time span post-administration in different calendar periods (e.g. differences might exist in terms of individual risk of exposure to SARS-CoV-2 among people who choose to get their booster dose at different times) [19].

Because of differences in the study design (e.g. study population, outcome and exposure definitions), the results of our study are difficult to compare with findings from the few peer-reviewed studies that evaluated the effectiveness of mRNA vaccines against the Omicron XBB sublineages [4-7]. One study conducted in the United States compared persons 65 years and above who had received a booster with bivalent COVID-19 mRNA vaccines with those who had received two to four monovalent vaccine doses a median of 13 months earlier [4]. The authors found that rVE against symptomatic infection likely caused by the Omicron XBB/XBB.1.5 sublineages was 43% in the time interval 15–90 days post-administration, close to the 46% rVE that we estimated in the time interval 15–60 days. They also observed no substantial differences between rVE against symptomatic infection related to Omicron BA.5 and that related to XBB/XBB.1.5 subvariants.

Although, to our knowledge, this is one of the largest studies focusing on rVE against severe COVID-19 due the Omicron XBB subvariant, it has some limitations. Firstly, as for other observational studies, although the analysis was adjusted for several covariates, a residual bias because of uncontrolled confounders might have affected our estimates. Secondly, under-reporting because of missed notification of self-diagnosed or unascertained cases has likely caused an overestimation of the number of susceptible people exposed to the risk of severe COVID-19, especially in the comparator group (first booster of an mRNA vaccine received at least 120 days earlier) [20]. This could have led to underestimate rVE, especially at later time intervals, even in the event that the proportion of under-reported cases did not differ between the compared groups. Thirdly, the study was conducted under the assumption that all cases included in the analysis were due to the Omicron XBB.1.5 or other XBB sublineages, estimated as predominating during the study period (88%) [14,15]. However, a small proportion of these cases could have been caused by other SARS-CoV-2 variants, possibly leading to an overestimation of rVE. Finally, since the overall impact of a monovalent vaccine matched to the circulating recombinant strains on both mild and severe cases cannot be predicted, the data of this study should be interpreted with caution in designing public health strategies.

## Conclusions

The results of this study suggest that, in persons aged 60 years and above, during predominant circulation of the Omicron XBB.1.5 and other XBB sublineages, a second or third booster of the Comirnaty bivalent Original/Omicron BA.4-5 mRNA vaccine, relative to a first booster received at least 120 days earlier, conferred additional protection against severe COVID-19 (rVE from 46% in the first two months to 14% in the 6 to 9 months interval post-administration). With a view to the upcoming autumn 2023 vaccination campaigns, in case of an early epidemic wave, the use of the bivalent Original/Omicron BA.4-5 mRNA vaccine might still be warranted until the recommended new monovalent COVID-19 vaccines targeting the Omicron XBB.1 descendent lineages become available.

## Supplementary Data

206000 Supplement
