# Supplementary material for: Relative effectiveness of bivalent Original/Omicron BA.4-5 mRNA vaccine in preventing severe COVID-19 in persons 60 years and above during SARS-CoV-2 Omicron XBB.1.5 and other XBB sublineages circulation, Italy, April to June 2023
Source: Euro Surveill. 2023 Aug 10;28(32):2300397. doi: 10.2807/1560-7917.ES.2023.28.32.2300397 (PMC10416574; doi:10.2807/1560-7917.ES.2023.28.32.2300397)
Supplement: Supplement [file 23-00397_FABIANI_SUPPLEMENT.pdf]

This supplementary material is hosted by Eurosurveillance as supporting information alongside the article *Relative effectiveness of bivalent Original/Omicron BA.4-5 mRNA vaccine in preventing severe COVID-19 in persons 60 years and above during SARS-CoV-2 Omicron XBB.1.5 and other XBB sublineages circulation, Italy, April to June 2023*, on behalf of the authors, who remain responsible for the accuracy and appropriateness of the content. The same standards for ethics, copyright, attributions and permissions as for the article apply. Supplements are not edited by Eurosurveillance and the journal is not responsible for the maintenance of any links or email addresses provided therein.

SUPPLEMENTARY MATERIAL

TABLE OF CONTENTS

	Page
**Supplementary Method S1**. Imputation of the expected date of death for causes unrelated to COVID-19 for vaccinated individuals who did not receive a diagnosis of SARS-CoV-2 during the study period (3 April to 4 June 2023).	3
**Supplementary Table S1**. Health-risk conditions recorded into the national vaccination registry.	4

Supplementary Methods S1. Imputation of the expected date of death for causes unrelated to COVID-19 for vaccinated individuals who did not receive a diagnosis of SARS-CoV-2 during the study period (3 April to 4 June 2023).

Mortality for causes unrelated to COVID-19 might be of some relevance in persons ≥60 years of age. In both the surveillance and vaccination databases, we have no information on the occurrence of such deaths. In the surveillance system, the notified cases are followed at least four weeks after infection to ascertain possible deaths due to COVID-19. A possible bias might be introduced in the analysis if considering alive and exposed to the risk of infection the persons who had not a diagnosis of SARS-CoV-2 infection during the study period but died for causes unrelated to COVID-19 before or during it (the risk of COVID-19-unrelated death might differ between the 1st booster and the 2nd booster recipients as people at higher risk, such as those aged 80+ years, are more likely to have received a 2nd booster). Therefore, to limit this potential bias toward overestimation of the relative vaccine effectiveness, assuming the age-sex-region specific risks of death in the year 2019 (when SARS-COV-2 was not circulating in Italy) as a proxy of the risk of death for causes unrelated to COVID-19 in the study period, we imputed the expected date of death following the last known survival date (the latest between the date of last vaccine dose administration or 29 days after the last notified infection before the study start, considering 28 days the maximum length of follow-up post-infection to ascertain a possible COVID-19 associated death in the surveillance system). The expected date of death for causes unrelated to COVID-19 was then used to exclude observations (those estimated to have died before the study starting date) or to censor them during follow-up.

We used the life tables by region, age, and sex for the year 2019, published by the Italian Institute of Statistics (http://dati.istat.it/Index.aspx?DataSetCode=DCIS_MORTALITA1), reporting the yearly probability of death $\mathbf{q_{x,y,z}}$ (per 1000), where x,y, and z indicate the region, age and sex, respectively.

Based on a uniform distribution, we randomly extracted and assigned a number in the interval 0-1 to every individual who did not receive a diagnosis of SARS-CoV-2 infection after the start of the study (3 April 2023), assuming it as the cumulative probability of death **S(t)**.

Based on the survival exponential function

$$\mathbf{S(t) = \exp(-q_{x,y,z} * t)},$$

we then calculated for each of these individual the expected number of survival days after the last known surviving date (i.e, the latest between the date of last vaccine dose administration or 29 days after the last infection date) as:

$$\mathbf{t = -\log[S(t)]/[q_{x,y,z}/(365*1000)]}.$$

Among the 16,160,986 vaccinated individuals ≥60 years of age who received at least one vaccine dose before 4 June 2023, we imputed a total of 552,790 (3.42%) deaths estimated to be occurred before the end of follow-up (4 June 2023) for causes unrelated to COVID-19.

Supplementary Table S1. Health-risk conditions recorded into the national vaccination registry.

**Description**
Cystic fibrosis
Defects of the complement system. Other specified disorders involving the immune mechanism; Deficiency or dysfunction of a single component (C1-C9)
Human immunodeficiency virus [HIV] disease, Human immunodeficiency virus, type 2 [HIV-2], Asymptomatic human immunodeficiency virus [HIV] infection status
Disorders involving the immune mechanism
Chronic Alcohol Misuse
Functional or anatomic asplenia
COPD
Chemotherapy or Radiotherapy
Coagulopathies
Diabetes Mellitus and other endocrinopathies
Patients in hemodialysis or with chronic kidney diseases expected to start dialysis
Hemoglobinopathy such as sickle cell anemia or thalassemia
Chronic Liver Disease
Cochlear implant
Chronic Kidney Disease
Chronic eczema or psoriasis
Diseases associated with a high risk of aspiration pneumonia
Chronic Cardiovascular Disease
Chronic Respiratory Disease
Motor neuron diseases
Chronic inflammatory diseases and malabsorption syndromes
Blood cancers (leukemia, lymphoma and myeloma)
Solid tumors
Obesity
Bone marrow transplant
Drug Misuse
Solid organ transplant
Patients with CSF leak from trauma or intervention
Patients in immunosuppressive treatment
Metabolic diseases
Hematopoietic diseases
Pathologies that require important surgical interventions
Neurological diseases
Cerebrovascular diseases
Down Syndrome
Disabilities (physical, sensorial, learning or psychic)
